# Development of Novel CD47-Specific ADCs Possessing High Potency Against Non-Small Cell Lung Cancer *in vitro* and *in vivo*

**DOI:** 10.3389/fonc.2022.857927

**Published:** 2022-05-12

**Authors:** Zu-Chian Chiang, Shubin Fang, Yang-kun Shen, Dongya Cui, Huanjiao Weng, Dawei Wang, Yuxiang Zhao, Jizhen Lin, Qi Chen

**Affiliations:** ^1^ Fujian Key Laboratory of Innate Immune Biology, Biomedical Research Center of South China, College of Life Science, Fujian Normal University, Fuzhou, China; ^2^ The Cancer Center, Union Hospital, Fujian Medical University, Fuzhou, China; ^3^ College of Photonic and Electronic Engineering, Fujian Normal University, Fuzhou, China; ^4^ The Department of Otolaryngology, Head and Neck Surgery, University of Minnesota Medical School, Minneapolis, MN, United States

**Keywords:** immunotoxin, antibody-drug conjugates, CD47 antigen, non-small cell lung cancer, macrophage, phagocytosis, targeted therapy

## Abstract

Targeted therapies hold promise for efficiently and accurately delivering cytotoxic drugs directly to tumor tissue to exert anticancer effects. CD47 is a membrane protein expressed in a variety of malignant tumors and hematopoietic cells, which plays a key role in immune escape and tumor progression. Although CD47 immunocheckpoint therapy has been developed in recent years, many patients cannot benefit from it because of its low efficiency. To strengthen and extend the therapeutic efficacy of anti-CD47 monoclonal antibody (mAb), we used the newly developed 7DC2 and 7DC4 mAbs as the targeting payload adaptor and VCMMAE as the toxin payload to construct novel CD47-specific immunotoxin (7DC-VCMMAE) by engineering cysteine residues. These CD47-specific ADCs have the better cell penetration, excellent DAR, similar payload distribution and good antigen-binding affinity. *In vitro*, 7DC-VCMMAE treatment induced death of non-small cell lung cancer (NSCLC) cell lines 95D and SPC-A1, but not A549 that express low levels of CD47 on the cell membrane. This finding suggests that 7DC-VCMMAE may possess greater therapeutic effect on NSCLC tumors expressing a high level of CD47 antigen; however, 7DC-VCMMAE treatment also promoted phagocytosis of A549 cells by macrophages. *In vivo*, 7DC-VCMMAE treatment had remarkable antitumor effects in a NSCLC cell line-derived xenograft (CDX) mouse model based on nonobese diabetic/severe combined immunodeficient (NOD/SCID). In summary, this study combined VCMMAE with anti-CD47 mAbs, emphasizing a novel and promising immunotherapy method for direct killing of NSCLC, which provides a valuable new way to meet the needs of the cancer therapy field.

## Introduction

Lung cancer is the leading cause of cancer mortality worldwide, with approximately 2.5 million new cases and 1.5 million deaths per year (1). Non-small cell lung cancer (NSCLC) accounts for approximately 85% of all lung cancer cases. The 5-year overall survival (OS) rate of NSCLC is less than 21% (2, 3). Antibody and chemotherapy treatments, as well as the development of tyrosine kinase inhibitors (TKIs), have improved the response rate and OS in patients with NSCLC (4, 5), but fewer than 20% of patients receive TKIs. Thus, the prognosis for advanced NSCLC remains poor (6, 7). Approximately 45% of lung cancers are classified as “cold tumors” with little or no infiltration of immune cells, which greatly reduces the efficacy of immunotherapy. Immune checkpoint inhibitors, such as anti-PD-1 antibodies, have only a 25% efficacy in advanced NSCLC. Therefore, there is a significant need for more effective therapeutics for NSCLC, particularly those that can target cold tumors.

Tumor-associated macrophages (TAMs) have been investigated as a potential immunotherapeutic strategy (8) because they promote the activation of immune cells and clearance of tumor cells through phagocytosis (9). Cluster of differentiation 47 (CD47) is a transmembrane glycoprotein with numerous functions (10), including acting as a “don’t-eat-me” signal to prevent phagocytosis (11). CD47 expression is widely distributed in hematopoietic cells and protects normal cells from phagocytosis by binding to an immunoglobulin-like cell surface receptor on macrophages, the signal regulatory protein alpha (SIRPα) (12, 13). Tumor cells, such as esophageal squamous cell carcinoma, also express CD47 (14, 15), which allows evasion of host immune surveillance and protection against phagocytosis (16–18). Overexpression of CD47 has been described in various malignancies, including leukemia (19, 20), lymphoma (21), multiple myeloma (22) and solid tumors, such as breast (23), colon (24), hepatocellular carcinoma (25), melanoma (26), and small cell lung cancer (27). CD47 is also highly expressed in NSCLC cells (28, 29) and primary NSCLC tumors, and promotes the invasion and metastasis of NSCLC (30). Therefore, targeting CD47 may provide a new option for targeting therapeutics to NSCLC.

Some studies have examined the potential of CD47 as an anti-cancer therapeutic target to prevent immune evasion of tumor cells (28); however, the CD47-targeted therapies tested thus far have shown low efficacy and limited benefit. Immunotherapeutic efficacy is related to the degree of infiltration of immune cells into the tumor tissue; for cold tumors, new therapeutic approaches are needed, which do not depend on immune cell infiltration. We hypothesized that CD47-targeted therapy could be improved through the development of anti-CD47 antibody-drug conjugates (ADCs). ADCs are one of the fastest developing classes of anticancer drugs; in recent years, they have been shown to efficiently and accurately deliver cytotoxic drugs directly to tumor tissue to exert anticancer effects and reduce systemic exposure and toxicity (31–33). ADCs comprise a monoclonal antibody (mAb) conjugated to small cytotoxic drugs *via* a chemical linker; the mAb delivers the drug to cancer cells that express the specific cell surface target antigen. Internalization of the mAb and release of the cytotoxic payload kills the cancer cell, though some studies have demonstrated that non-internalized ADC products can release the cytotoxic drug into the tumor microenvironment to elicit a potent therapeutic effect (34). Since 2000, ADC drugs have attracted more and more attention from the pharmaceutical industry. So far, more than 100 ADC drugs are undergoing development and 5 ADCs drugs have been approved by FDA. There are at least 5 VC-linked MMAE (VCMMAE) ADC drugs developed globally (35). Recently, an ADC drug targeting CD47 has emerged by using Sulfo-SMCC linker to make non-cleavable ADC, namely anti-CD47-DM1 (36). However, peptide-based linkers are stable in unsuitable pH condition and different serum protease inhibitors; therefore, these peptide linkers are stable in the systemic circulation and only unleash the drug in the target cells (37). Valine citrulline (V-C) is the most commonly used peptide linker in current clinical research. One example of successful use of the V-C linker in ADC design is the Adcetris^®^ for targeting CD30 that has been approved by FDA (38). 

In this study, we developed CD47-specific ADCs *via* V-C linker as a new targeted therapy for NSCLC. Firstly, the spleen cells of mice sensitized by CD47 antigen were collected and sequenced on a large scale, and the phage display technology was used to screen the anti-CD47 antibody with high affinity and specificity. Then, we established CD47-targeted ADCs as a specific targeted drug. Through further identification and characterization, the specific-CD47 ADC drug was described. Finally, the killing effect and phagocytosis induction effect on NSCLC *in vitro* were evaluated, and the antitumor efficacies on NSCLC *in vivo* were confirmed by NSCLC cell-derived xenograft (CDX) mouse model, using NOD/SCID mice to mimic the cold tumor environment.

## Materials and Methods

### Chemicals and Reagents

Ammonium sulfate, sodium phosphate, sodium chloride, Tris (2-carboxyethyl) phosphine (TCEP), N-acetylcysteine (NAC), isopropyl alcohol (IPA), dimethyl sulfoxide (DMSO), 2-mercaptoethanol (2-ME), ethylenediaminetetraacetic acid (EDTA), phosphate buffered saline (PBS), and TWEEN^®^ 20 were purchased from Sigma-Aldrich. Centrifugal filter tubes (Amicon-30 kDa) were purchased from Merck Millipore. Maleimidocaproyl-valine-citrulline-monomethyl auristatin E (VCMMAE) was obtained from MedChem Express. Lithium Dodecyl Sulfate (LDS) sample loading buffer (4X) and 12% acrylamide of PAGE gel were purchased from Thermo Fisher Scientific. Grener F 96-well immunoplates were purchased from Sigma-Aldrich.

### Isolation of Mouse Anti-Human CD47 Monoclonal Antibodies from B Cells of Immunized Mice

To isolate CD47 specific monoclonal antibodies, 2 x 10^7^ the mouse lymphocytes were collected from the spleen of 3 mice immunized with the human CD47 antigen. All B cells were collected and pooled from the mouse spleens. A mouse single strand fragment variable (scFv) library with high-quality was constructed by using approximate 10^7^ the mouse lymphocytes following the method described previously (39, 40). Specifically, the total RNA of the B cells was extracted by conventional Trizol reagent (41), the immune scFv library (containing approximate 10 million different antibodies) were constructed and displayed by phage. The human CD47 antigen was used to screen specifically bound antibodies and isolated from the library through three consecutive enrichment steps. The ability of clones produced by these enrichment steps to bind human CD47 was tested by ELISA. Two clones, namely 7DC2 and 7DC4, were selected. The specific chimeric CD47 binding antibodies containing the mouse Fab plus human IgG1 Fc fragment were constructed, and were expressed, purified and used for later toxin conjugation and further analyses.

### Preparation of the New Anti-CD47 Antibodies, 7DC2 and 7DC4

Expi293 (Gibco), a high-yield transient expression system based on suspension-adapted Human Embryonic Kidney (HEK) cells, was used as a production host for 7DC monoclonal antibody expression. OPM-293 CD03 medium (Shanghai OPM Biosciences) is a chemically defined, serum-free, protein-free medium for growth and transfection of Expi293 cells. OPM-293 CD03 medium was supplemented with 2 mM L-glutamine (Gibco) before use. Expi293 cells were incubated in a 37°C incubator with 80% relative humidity and 5% CO_2_ on an orbital shaker platform (Thermo fisher). Glucose (SINOPHARM) and OPM-CHO PFF05 (Shanghai OPM Biosciences) were added as feed.

After thawing, Expi293 cells were subcultured to 0.3 × 10^6^ - 0.5 × 10^6^ cells/mL every 4-5 days in suspension in a 125 mL shaker flask containing 30 mL OPM-293 CD03 medium. Before transfection, 20 μg light chain plasmids and 10 μg heavy chain plasmids (endotoxin-free) were mixed with PEI transfection reagent, and then the DNA-PEI complexes were added to transfect cells in the shaker flask. Feed medium was added every 2 days after transfection. 300 g/L glucose was added once daily to achieve a residual glucose concentration of 1 g/L. Glucose concentration was determined using a Glucose Assay Kit (Shanghai Rongsheng bio). When the viability was lower than 75%, cell cultures were harvested.

### Flow Cytometry

Lung cancer cell lines SPC-A-1 (FH0082, Shanghai Fuheng Biological Technology Co., Ltd.), A549 (CL-0016, Procell Life Science&Technology Co., Ltd.), and 95D (CL-0011, Procell Life Science&Technology Co., Ltd.) (1 × 10^6^), authenticated by STR and Amelogenin analysis (hppt://web.expasy.org/cellosaurus-str-search) were placed in a 1.5 ml centrifuge tube, centrifuged at 1,200 rpm/min for 5 minutes, and the supernatant was discarded. Cells were suspended in 100 µl FACS buffer and 1 μl of anti-human CD47 antibody with PE fluorescence (clone: CC2C6, BioLegend, Inc.) was added, followed by incubation for 30 min at 25°C. After staining, the cells were washed twice with flow buffer (PBS + 2% FBS). Antibodies with PE fluorescence not bound to cells were removed by centrifuging at 1,200 rpm/min for 5 minutes to discard the supernatant. The cells were resuspended in 300 μl of flow buffer and the fluorescence intensity was analyzed using a FACSymphony™A5 (BD Biosciences).

Antibodies (7DC) and ADCs (7DC-VCMMAE) we developed also were used to check the CD47 expressions of three cancer cell lines. The cells (1 × 10^6^) were firstly incubated by 2 μg of 7DC2, 7DC4, 7DC2-VCMMAE and 7DC4-VCMMAE for 30 min, respectively. After washing, a Goat anti-Human IgG Fc secondary antibody labeled PE (eBioscience™, Invitrogen) was added and incubated for 30 min. They were washed again and centrifuged, and the supernatant was discarded. The secondary Antibody labeled with PE was used to directly bind with the cells without 7DC or 7DC-VCMMAE as a negative control. Their Mean Fluorescence Intensity (MFI) was analyzed as described above.

### Internalization Assay for 7DC2 and 7DC4

SPC-A-1 cells were suspended at 2 × 10^5^ cells per mL and treated with 7DC2 and 7DC4, labelled with Fluor-488 (LinKine™ AbFluor 488 Labeling Kit, Abbkine) according to the manufacturer’s protocol, at the concentration of 5 μg/mL in complete medium. After 4-hour incubation at 37°C with 5% CO_2_, the cells were washed once in PBS (pH 7.4) to remove unbound antibodies. DAPI was used to stain the nucleus and antibody-free Fluor-488 as a negative control. Cells were washed again and subsequently imaged with confocal microscopy. Fluorescence images were acquired with a Zeiss Axio Observer Z1 microscope with laser scanning unit LSM 780 fitted with a Axio Cam MRm camera.

### Development of the New ADCs, 7DC2-VCMMAE and 7DC4-VCMMAE

**Scheme 1:**
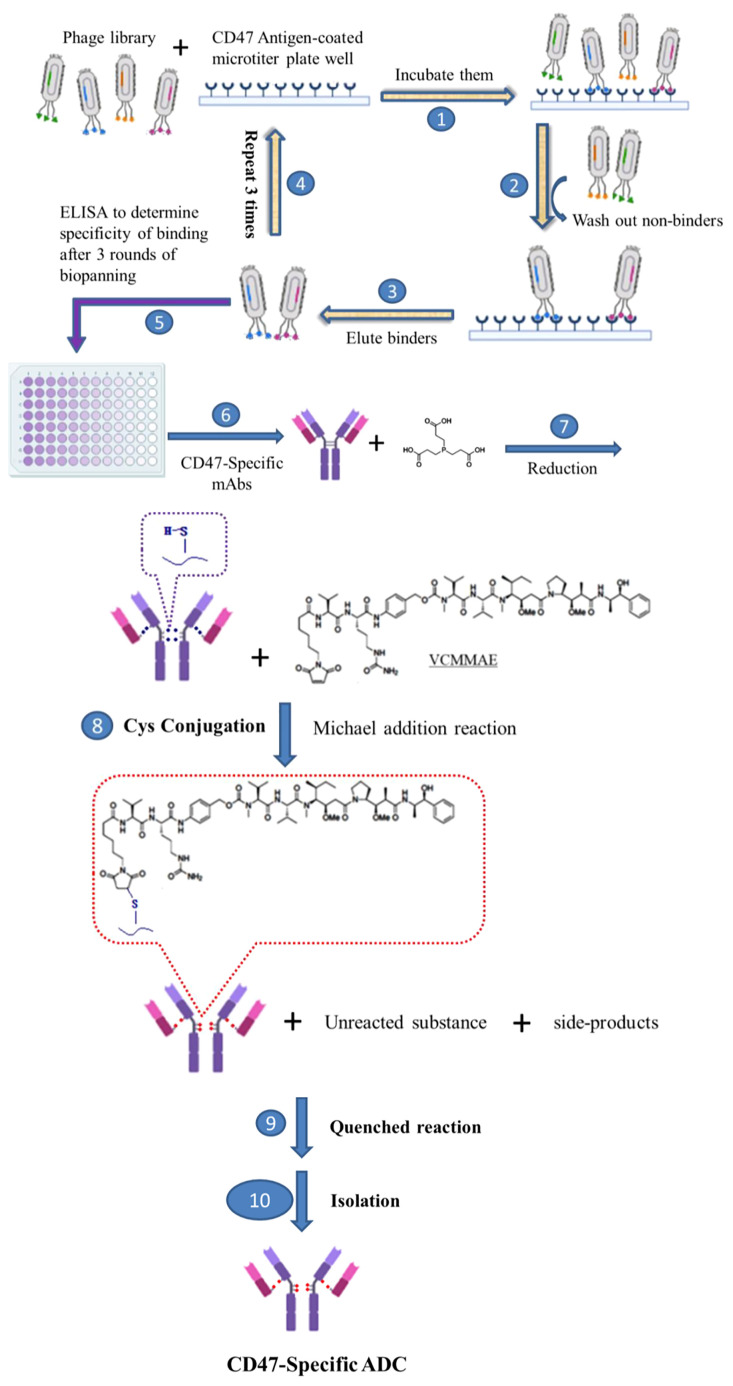
Development of novel CD47-specific ADCs. The specific CD47 mAb, 7DC, was discovered by phage display shown in steps 1. incubating them, 2. washing out non-binders, 3. eluting binders, 4. repeating steps from 1 to 3 for 3 rounds, 5. determining specific of binding by ELISA and 6. making CD47-specific mAbs. CD47-specific ADCs, 7DC2-VCMMAE and 7DC4-VCMMAE, were established by conjugating the small molecule cytotoxic drug, VCMMAE, to the IgG linker region through a series of chemical reactions, as shown in steps 7. reducing the disulfide bonds of CD47-specific mAb to make free sulfhydryl groups, 8. Cys conjugation by Michael addition reaction, 9. quenched reaction, 10. isolation of CD47-specific ADC.

After identification of the CD47-specific mAb, 7DC, CD47-specific ADCs (7DC2-VCMMAE and 7DC4-VCMMAE) were established in a series of chemical reactions, as illustrated in **Scheme 1**. MMAE with maleimide-modified VC was conjugated to the 7DC2 and 7DC4 monoclonal antibody by Michael addition to form 7DC2-VCMMAE and 7DC4-VCMMAE, respectively. The pH 6.8 conjugation buffer solution contained 50 mM sodium phosphate, 50 mM sodium chloride and 2 mM EDTA. The 7DC2 and 7DC4 solution were incubated with conjugation buffer solution respectively, and were filtered by using Amicon-30 kDa. 13.5 μM of 7DC2 and 7DC4 were used in a reduction reaction with excess Tris (2-carboxyethyl) phosphine (TECP, Sigma-Aldrich) at 30°C for 2 hours to create free sulfhydryl groups.

The samples were then conjugated with 10.8 equivalents of VCMMAE dissolved in DMSO at 30°C to achieve high drug-to-antibody ratio (DAR) and drug distributions. The reaction time was controlled for 12 hours. After reaction quenching, the unreacted VCMMAE and side-products were removed by Amicon-30 kDa using 10.8 equivalents of N-Acetylcysteine (NAC). Finally, Amicon-30 kDa was used again to purify the products; finished products were stored at 4°C for later analysis and application in experiments.

### UV-VIS Photo-Profiling

UV-VIS photo-profiling was employed to rapidly confirm successful preparation of ADCs. Nanodrop 2000 spectrophotometers were used to measure the UV-VIS photo-profile of ADCs. The maximum absorption wavelengths of the samples were selected at 280 nm and 248 nm to detect the spectral changes before and after antibody conjugation with VCMMAE. Two microliters of each sample (7DC2, 7DC4, 7DC2-VCMMAE, and 7DC4-VCMMAE) was taken for UV-VIS photo-profiling under the above described conditions.

### LDS-PAGE Analysis of ADCs

Polyacrylamide gel electrophoresis (PAGE) of 7CD2, 7DC2-VCMMAE, 7CD4, and 7DC4-VCMMAE was conducted under reducing and non-reducing conditions. Lithium dodecyl sulfate (LDS) sample loading buffer (4X) and working solution, containing 106 mM Tris HCl, 141 mM Tris base, 2% LDS, 10% glycerol, 0.51 mM EDTA, 0.22 mM G250 Coomassie Blue, and 0.175 mM phenol red, pH 8.5 with and without 2-mercaptoethanol (2-ME), were used to run the samples. Samples (approximately 4 μg of protein per sample) were denatured at 95°C for 15 minutes. The mixtures were loaded on a 12% polyacrylamide gel and then were separated at 150 V/160 mA for approximately 1 hour. After electrophoresis, the gel was stained with InstantBlue™ solution (Bio-Rad, Hercules, CA), then destained overnight in ultrapure water. Gel images were captured by a Gel Doc™ XR^+^ (BIO-RAD).

### Binding Affinities of the ADCs Determined by ELISA

The half-maximal effective concentrations (EC_50_ values) of 7CD2, 7DC2-VCMMAE, 7CD4, and 7DC4-VCMMAE were determined by titrating IgG antibodies on immobilized CD47/ECD (Gln 19 - Pro 139, His Tag, ACROBiosystems) is expressed from human 293 cells (HEK293). It contains AA (Accession # NP_942088). with ELISA. In brief, CD47/ECD antigen (0.2 μg per well) in PBS buffer (pH 7.4) was coated on Grener F 96-well immunoplates for 16 hours at 4°C, and then the wells were blocked with 2% BSA in PBST (phosphate-buffered saline with 0.05% Tween-20) for 1.5 hours. Samples in PBST with 0.5% BSA were prepared at 11 concentrations by performing two-fold serial dilutions. After blocking, 100 μl of each diluted sample was added to each well and incubated for 1 hour with gentle shaking. The plate was washed with 300 μl of PBST 4 times, and then 100 μl of horseradish peroxidase/anti-human IgG antibody conjugate (1000X dilution) in PBST with 0.5% BSA was added and incubated for 1 hour at room temperature. After washing 4 times with PBST buffer and twice with PBS, the samples were treated with 3,3’,5,5’-tetramethylbenzidine peroxidase substrate for 3 minutes, quenched with 1.0 M HCl, and measured at 450 nm with an ELISA reader. The EC_50_ (ng/ml) was calculated by ED_50_ Plus v1.0 software.

### HIC-HPLC Analysis

Characterization of DAR and drug distribution was accomplished by using hydrophobic interaction chromatography-high performance liquid chromatography (HIC-HPLC). A TSKgel Butyl-NPR column (Tosoh Bioscience) column with 2.5 μm particles and 4.6 mm ID × 3.5 cm lengths was employed.

Mobile phase A, an aqueous solution of 1.8 M ammonium sulfate with 25 mM sodium phosphate at pH 7, and mobile phase B, a mixture of 75% (v/v) aqueous solution of 25 mM sodium phosphate at pH 7 with 25% (v/v) isopropyl alcohol, were generated to elute the samples. The analytical method was established by the linear gradient from 100% buffer A to 100% buffer B for 12 minutes, a flow rate at 1 mL/min and the temperature at 25°C. The samples were monitored by a UV detector at 248 nm.

The DAR of the samples was calculated by


(1) $$\text{DAR}=\;\sum \text{n}\times {\text{A}}_{\text{n}}/\sum {\text{A}}_{\text{n}}$$

Where n denotes the number of drugs attached to the antibody (DAR species) and A_n_ denotes the area under each DAR species peak cluster.

### Cell Culture and Cytotoxicity Assays

Human lung cancer cell lines (95D, A549, and SPC-A-1) were grown in RPMI 1640 and DMEM media (HyClone) supplemented with 10% fetal bovine serum at 37°C in a humidified atmosphere containing 5% carbon dioxide. 95D, SPC-A-1, and A549 cells were seeded at densities of 2 × 10^4^ cells/well in 96-well plates. After cell attachment to the well, 5-fold serial dilutions of the anti-CD47 antibodies (7DC) and ADCs (7DC-VCMMAE) were added, and the cells were incubated at 37°C for 2 to 3 days. To evaluate cytotoxic activity, relative cell viability was measured using the WST-1 colorimetric assay (Roche) following the manufacturer’s instructions.

### *In Vitro* Phagocytosis Assay

To demonstrate the phagocytosis induction effect of CD47-specific ADCs *in vitro*, Raw 264.7 macrophages were co-cultured with A549, SPC-A-1, or 95D cancer cells. The phagocytosis phenomenon can be directly observed and compared through the fluorescence imaging method according to the method of Willingham et al. (28) and slightly modified. In brief, 1 × 10^5^ macrophages were plated per well in a 6-well tissue culture plate. The cancer cells were labeled with CFDA SE according to the manufacturer’s protocol. Macrophages were incubated in serum-free medium for 2 hours before the addition of 2 × 10^5^ CFDA SE-labeled the cancer cells. Anti-CD47 antibodies and the ADCs were added and incubated at 40 nM for 2.5 hours at 37°C. Macrophages were repeatedly washed and subsequently imaged with an inverted microscope.

### *In Vivo* Tumor Xenograft Studies

95D cells, a human highly metastatic lung cancer cell line, were grown in RPMI 1640 medium (Gibco) as described above. All mouse experiments were conducted according to guidelines and experimental protocols approved by the Institutional Animal Care and Utilization Committee (IACUC) of Fujian Normal University (Protocol ID: 20200010). The 95D cell line-derived xenograft model (NSCLC CDX- model) was established by subcutaneously inoculating 1 × 10^6^ cells into the right flank of 6-week-old female NOD.CB17-Prkdcscid/NcrCrlBltw NOD/SCID mice (WSSYDW). When the tumors reached suitable tumor size 14 days after inoculation, the mice were randomly assigned into six groups (four mice per group): 7DC2 (20 mg/kg), 7DC4 (20 mg/kg), 7DC2-VCMMAE (20 mg/kg), 7DC4-VCMMAE (20 mg/kg), free VCMMAE (0.1755 mg/kg), and PBS (200 μl). All groups’ mice were administered without anesthesia. The 95D tumor-bearing mice were treated once a week for a total of three doses for each group through intraperitoneal injection. Tumor size and weight change in mice were recorded twice a week. Tumor volume was calculated by using the ellipsoid formula: length × width × height × 0.523. Survival probability over time was evaluated by the Kaplan-Meier method; mice with tumor of the size above 200 mm^3^ were considered treatment failures and were removed from the surviving population when calculating the Kaplan-Meier curves. Thus, “survival” in this study was defined as mice that were alive and with tumor burden less than 200 mm^3^.

### Serum Biochemical Analysis

8-week-old female NOD/SCID mice were intraperitoneally injected with 20 mg/kg of 7DC2, 7DC4, 7DC2-VCMMAE, or 7DC4-VCMMAE; 133.5 nmole/kg of VCMMAE; or 10 ml/kg of PBS once a week for a total of three doses. Blood samples were collected and assayed for alanine transaminase (ALT), alkaline phosphatase (ALP), creatinine (CRE), and blood urea nitrogen (BUN) using Mindray BS-800 (Mindray) according to manufacturer’s instructions.

### DyLight 680 Conjugation, *in vivo* Optical Imaging, and *ex vivo* NIRF Imaging

The interchain disulfide bond of 7DC2 and 7DC4 were reduced by excess of TCEP at 30°C for 2 hours to produce free sulfhydryl groups. The samples were then conjugated with 10.8 equivalents of DyLight 680 (Thermo Scientific) at 30°C for 4 hours. Unreacted DyLight 680 was removed by using Dye Removal Column kit (Pierce) and conjugation efficiency was determined using Nanodrop (Thermo Scientific) to calculate the molar ratio of DyLight 680 to protein.

Mice bearing 95D tumors were intraperitoneally injected with 0.5 nmol of 7DC2-DyLight 680 or 7DC4-DyLight 680 (100 μL per injection; 3 mg/kg) or 1 nmol of free DyLight680 as a control group. For *in vivo* optical imaging, NIRF images were obtained at 24 hours using a small-animal IVIS imaging system (IVIS-Spectrum, Xenogen) with excitation and emission wavelengths of 675 and 720 nm. Fluorescence emission was normalized to photons per second per centimeter squared per steradian (p/s/cm2/sr). For *ex vivo* NIRF imaging, the mice were euthanized 24 hours after injection, and blood and organs were collected. NIRF images were acquired for each tissue as described above.

### Statistical Analysis

Data are expressed as mean ± SD and n ≥ 4 as indicated. Differences between two groups were analyzed by two-tailed Student’s t-test, and data set comparisons with P values of < 0.05 were considered statistically significant.

## Results

### CD47 Overexpression in Lung Cancer Cell Lines

**Figure 1:**
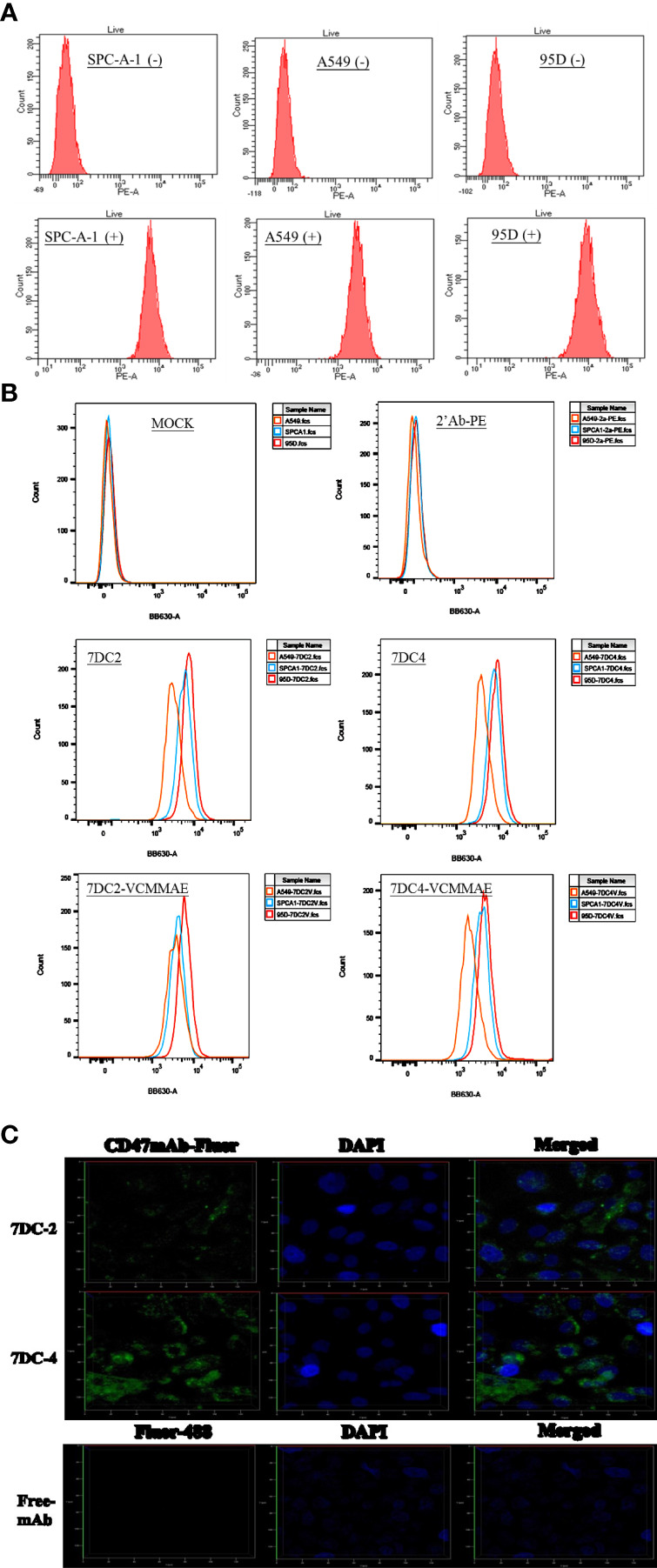
Characterization of CD47-overexpressing cancer cells and anti-CD47 antibodies. **(A)** Flow cytometry showing that CD47-positive human lung cancer cell lines A549, SPC-A-1, and 95D express different amounts of CD47 on their surfaces, stained by using anti-human CD47 antibody with PE fluorescence (clone: CC2C6). **(B)** Flow cytometry showing that the strength of CD47-expressed cell surface of three lung cancer cells were in the order of A549 < SPC-A-1 < 95D, stained by using 7DC2, 7DC4, 7DC4-VCMMAE, or 7DC4-VCMMAE and the anti-Human IgG Fc secondary antibody labeled with PE, respectively. **(C)** Confocal fluorescence images confirming that the anti-CD47 mAbs, 7DC2 and 7DC4, are internalized by SPC-A-1 lung cancer cells (green, Fluro-488-labelled mAbs; blue, DAPI nuclear staining).

SPC-A-1, A549, and 95D are human lung cancer cell lines with differences in aggressiveness, metastasis, drug resistance, and CD47 expression levels. To quantify CD47 expression at the cell surface, the three cell lines were incubated with anti-CD47 antibody labelled with phycoerythrin (PE) and flow cytometry was used to detect cell membrane CD47. All cells expressed CD47 protein on the cell membrane but at distinctly different levels (**Figure 1A**). CD47 overexpression was greatest in 95D cells, followed by SPC-A-1 and A549 cells (**Figure 1A** and **Table 1**). SPC-A-1 and 95D cells expressed approximately 2- to 3-fold more CD47 on the cell surface than A549 cells.

**Table 1:** Cell surface expression of CD47 on different lung cancer cell lines.

Population (Live Cells)	MFI (Mean)	MFI (Medium)
A549 (-)	44.4	41
A549 (+)	3058	2765
SPC-A-1 (-)	33.9	33
SPC-A-1 (+)	6126	5517
95D (-)	51.5	45
95D (+)	9456	8182

(+) indicates cells incubated with anti-CD47 antibody labeled with PE-A; (-) represents cells only (control). Events (Cell count) were 5000 cells per group for calculating. The colored values (mean fluorescence intensity, MFI).

In addition, three lung cancer cells were treated with 7DC2, 7DC4, 7DC2-VCMMAE, or 7DC4-VCMMAE and followed by staining with the anti-Human IgG Fc secondary antibody labeled with PE (**Figure 1B** and **Supplementary Table S1**). As a result, we found that the fluorescence intensity showed in the order of 95D > SPC-A-1 > A549 (**Figure 1B**; **Supplementary Table S1**). In other words, the expression of CD47 on the cell membrane of three lung cancer cells is in the order of 95D > SPC-A-1 > A549. This result is consistent with that in **Figure 1A** and **Table 1**. Additionally, in the absence of 7DC or 7DC-VCMME, the anti-Human IgG Fc secondary antibody labeled PE does not interact with cells, so the fluorescence intensity of those cells does not change.

### Internalization of CD47-Specific mAbs

Antibodies used for constructing ADCs must be able to enter tumor cells to deliver their cytotoxic payload. Using phage display screening of an internal antibody library, we identified 7DC2 and 7DC4 mAbs as two candidates that recognize different antigenic epitopes on the CD47 antigen. We examined whether the two mAbs are internalized into SPC-A-1 cells (moderate overexpression of cell surface CD47) after binding to CD47. 7DC2 and 7DC4 mAbs were labeled with Fluro-488 and incubated with SPC-A-1 cells. Confocal microscopy revealed fluorescence signals on the cell membrane and within the cell, suggesting that both 7DC2 and 7DC4 are able to enter the cell after binding to cell surface CD47 (**Figure 1C**).

### Rapid Identification of VCMMAE Binding to CD47 mAbs

We next identified cytotoxic small molecules that could successfully bind to our two candidate antibodies. We used UV-VIS spectrophotometry to rapidly screen conjugates based on the difference in optical properties between antibodies and cytotoxic drugs, resulting in a UV-VIS photo-profile (spectrogram) of the ADC.

**Figure 2:**
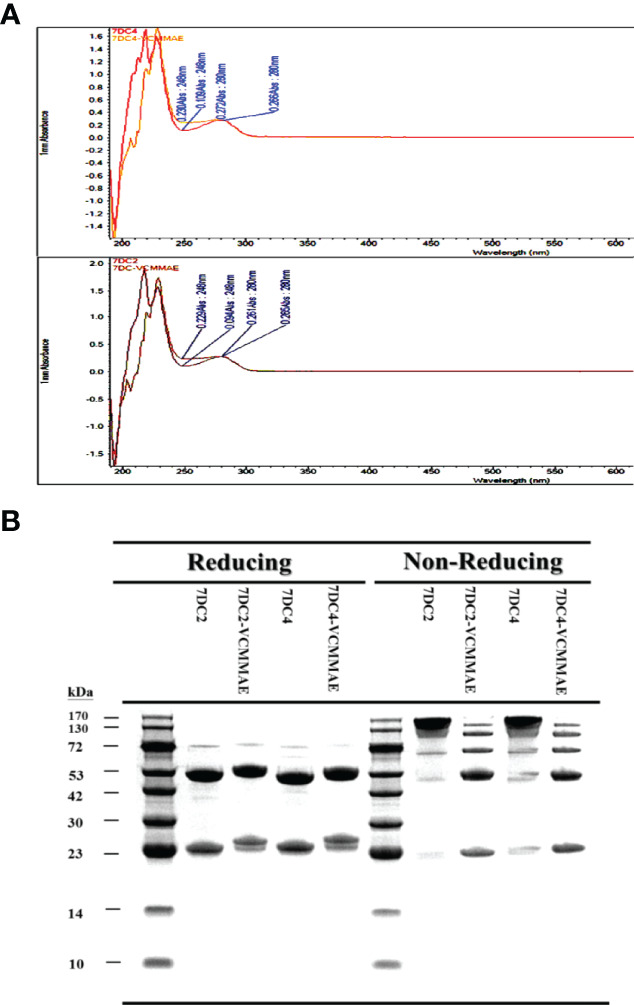
Characterization of 7DC2-VCMMAE and 7DC4-VCMMAE. **(A)** UV-VIS photo-profiles of both ADCs. After the conjugation of 7DC2 or 7DC4 mAbs with VCMMAE, their UV-VIS photo-profiles changed from a hill to flat appearance and in spectral ranges from 248 nm to 280 nm. **(B)** LDS-PAGE images of both ADCs under non-reducing and reducing conditions, confirming that both ADCs were successfully generated by Michael addition (see **Scheme 1**).

We found that the conjugation of either 7DC2 or 7DC4 mAb with valine-citrulline-monomethyl auristatin E (VCMMAE) led to a significant change in the UV-VIS photo-profile in the spectral range from 248 nm to 280 nm (**Figure 2A**). The absorption values ​​of 7DC4 and 7DC2 mAbs at 248 nm and 280 nm were 0.109 and 0.266, and 0.094 and 0.26, respectively. After the binding reaction, the values were 0.230 and 0.272, and 0.229 and 0.266, respectively (**Figure 2A**). Thus, the absorbance ratio of VCMMAE at 248 nm and 280 nm increased from 0.36 - 0.41 to 0.85 - 0.86, indicating successful bonding with 7DC2 or 7DC4 to form the ADCs 7DC2-VCMMAE and 7DC4-VCMMAE.

### Confirmation of ADC Formation by LDS-PAGE

We used LDS-PAGE to confirm the successful production of 7DC2-VCMMAE and 7DC4-VCMMAE; the different molecular weights of 7DC2, 7DC4, 7DC2-VCMMAE, and 7DC4-VCMMAE under reducing and non-reducing conditions are displayed in **Figure 2B**. An antibody is composed of two heavy chains and two light chains linked by four disulfide bonds. After reducing the disulfide bonds to free-sulfhydryl groups with 2-mercaptoethanol (2-ME), the antibody and ADC appeared as two main molecular bands: the lower band indicates the light chain and the higher band indicates the heavy chain (**Figure 2B**). The light chain of 7DC4 mAb had a slightly higher molecular weight than that of 7DC2 mAb, while the heavy chain of 7DC4 mAb had a slightly lower molecular weight than that of 7DC2 mAb. This may be caused by differences in glycosylation. When VCMMAE was bonded to either 7DC2 or 7DC4 mAb, the molecular weight of both the heavy and light chain increased significantly, with both bands shifting upwards.

Under non-reducing conditions (**Figure 2B**), both 7DC2 and 7DC4 mAbs displayed a main band of about 150 kDa, while 7DC2-VCMMAE and 7DC4-VCMMAE clearly showed five bands with molecular weights of about 25, 50, 75, 100 and 125 kDa. The band at 150 kDa almost disappeared, indicating that all four disulfide bonds were reduced to free-sulfhydryl groups so that VCMMAE could bind to the antibody through Michael addition to make special 8 DAR. When free-sulfhydryl groups reacted with VCMMAE, the molecular weight increased and each band shifted to a higher position. This result further demonstrated that VCMMAE was successfully bonded to 7DC2 and 7DC4 mAbs.

### Drug-to-Antibody Ratio and Distribution of 7DC-VCMMAE

**Figure 3:**
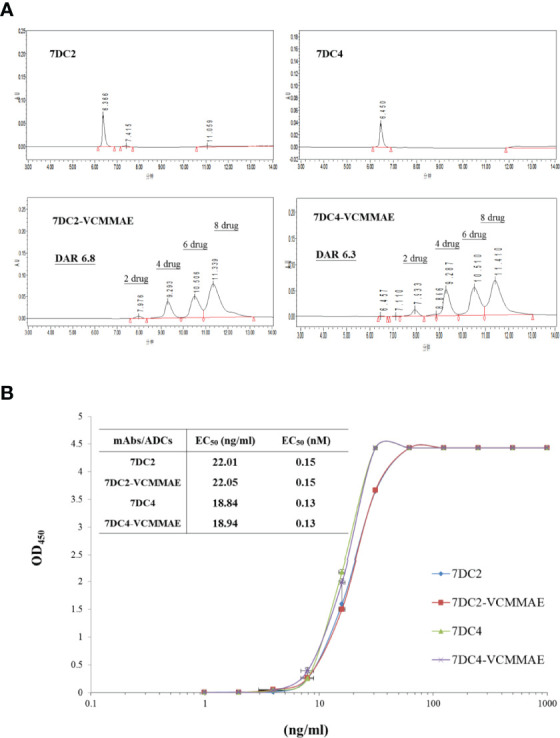
Drug-antibody ratio and drug distribution in 7DC2-VCMMAE and 7DC4-VCMMAE. **(A)** Representative HIC-HPLC chromatograms of both ADCs show similar DAR and drug distributions. **(B)** The EC_50_ values of the graded dose-response curve of both ADCs show similar antigen binding affinities (n = 3).

One of the key factors affecting antitumor efficacy of ADCs is the DAR and distribution of payload binding to given concentration of antibody. We used HIC-HPLC to evaluate the characteristics of 7DC2, 7DC4, 7DC2-VCMMAE, and 7DC4-VCMMAE (**Figure 3A**). 7DC2 or 7DC4 showed only a single absorption peak, demonstrating the purity and structural integrity of the antibodies, although a small number of antibodies did not contain four complete disulfide bonds (See **Figure 2B**, non-reducing conditions). We speculated that non-covalent bonds such as hydrogen bonds and hydrophobic bonds are formed in addition to disulfide bonds to stabilize the 3D structure of the antibody. The absorption peaks of 7DC2 and 7DC4 were displayed at the retention times 6.366 and 6.450 (min), respectively. This result indicates that these two antibodies have similar hydrophilic properties, although they have different amino acid sequences and different glycosylation sites.

**Figure 4:**
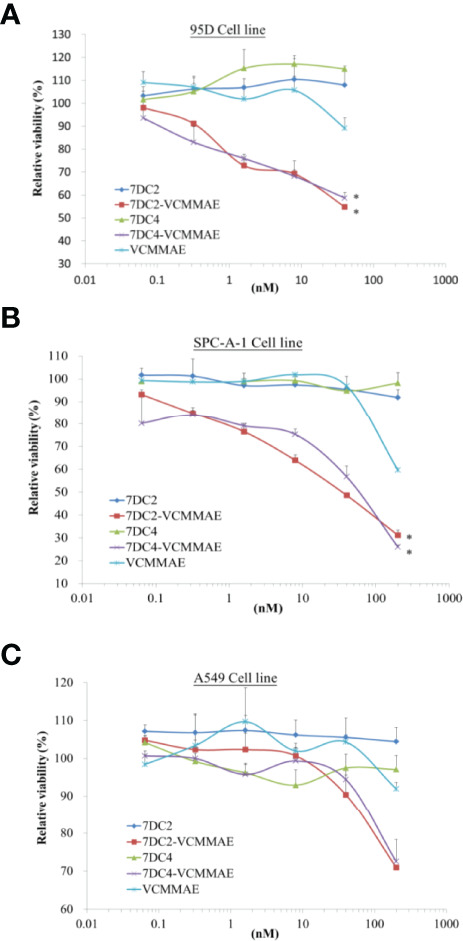
Cytotoxic activity of 7DC2 VCMMAE and 7DC4 VCMMAE in lung cancer cell lines. Three different lung cancer cell lines, **(A)** 95D, **(B)** SPC-A-1, and **(C)** A549, were treated with the indicated concentrations of 7DC2 VCMMAE or 7DC4 VCMMAE. Both ADCs induced significant cell death in 95D and SPC-A-1 cells, but showed lower efficacy in A549 cells. All experiments were performed in three replicates. * indicates P < 0.05.

The chromatogram of 7DC2-VCMMAE showed four absorption peaks at the retention times of 7.976, 9.293, 10.506, and 11.339 (min), but the absorption peak of the 7DC2 antibody (at retention time 6.366 min) was almost undetectable. Thus, all of 7DC2 reacted with VCMMAE but with a different distribution of the drug payload, labeled as 2, 4, 6, and 8 in **Figure 4A**. 7DC4-VCMMAE also exhibited four absorption peaks at the retention times 7.933, 9.287, 10.510, and 11.410 (min), and again, the 7DC4 antibody absorption peak (at retention time 6.450 min) was nearly absent. The drug distribution of 7DC4-VCMMAE was similar to that of 7DC2-VCMMAE, but with slightly different area ratios. Each drug distribution peak for 7DC2-VCMMAE had higher area ratios compared with those of 7DC4-VCMMAE. Therefore, the DAR of 7DC2-VCMMAE was slightly higher than that of 7DC4-VCMMAE (6.8 and 6.3, respectively).

### Antigen Binding Affinity of 7DC-VCMMAE

Another factor affecting the therapeutic efficacy of ADCs is antigen binding affinity, which depends on the structure of the antibody itself and whether its conformation changes after binding of the cytotoxic payload to form an ADC. ELISA was used to assess the binding affinity and EC_50_ of the ADCs to the CD47 antigen. The EC_50_ value and the graded dose-response curves of 7DC2 mAb, 7DC4 mAb, 7DC2-VCMMAE, and 7DC4-VCMMAE are shown in **Figure 3B**.

The EC_50_ values of 7DC2 and 7DC4 mAb binding to CD47 antigen were 0.15 nM (22.01 ng/ml) and 0.13 nM (18.84 ng/ml), respectively. Compared with 7DC4 mAb, the binding affinity of 7DC2 mAb was slightly but not significantly lower than that of 7DC4 mAb. The EC_50_ values of ADCs 7DC2-VCMMAE or 7DC4-VCMMAE were almost the same as those of the mAbs, at 0.15 nM (22.05 ng/ml) and 0.13 nM (18.94 ng/ml), respectively. Thus, binding of VCMMAE to form the ADC did not change the CD47 antigen binding affinity of either antibody.

### Cytotoxic Activity of 7DC-VCMMAE ADCs *In Vitro*

We used 95D, SPC-A-1, and A549 human lung cancer cell lines, each of which express different concentrations of cell surface CD47 antigens (see **Figure 1A**), to evaluate the cytotoxic effect of 7DC2-VCMMAE and 7DC4-VCMMAE *in vitro*, measured as the relative viability rate (**Figure 4**). 95D is a highly metastatic lung cancer cell line with high expression of CD47 glycoprotein. In a dose-response experiment, we treated 95D cells with 7DC2 mAb, 7DC4 mAb, VCMMAE, 7DC2-VCMMAE, or 7DC4-VCMMAE at 40, 8, 1.6, 0.32, and 0.064 nM. In the 7DC2 and 7DC4 mAb negative control groups, relative cell viability rates were almost the same regardless of mAb the concentration, indicating no cytotoxic activity (**Figure 4A**). In VCMMAE group, there was almost no difference in the relative cell viability rate up to 8 nM, which then dropped to 90% at a VCMMAE concentration of 40 nM. By contrast, the relative cell viability rates of 95D decreased with increasing concentrations of either 7DC2-VCMMAE or 7DC4-VCMMAE, reaching approximately 50% at the 40 nM concentration. The IC_50_ of both 7DC2-VCMMAE and 7DC4-VCMMAE was approximately 40 nM.

SPC-A-1 is a human lung adenocarcinoma cell with moderate expression of CD47 glycoprotein; thus, we increased our dose-response concentrations to a maximum of 200 nM (**Figure 4B**). As with 95D cells, a high concentration of 7DC2 or 7DC4 mAb did not affect the relative cell viability rate of SPC-A-1 cells, whereas the relative cell viability rate decreased sharply to approximately 60% with 200 nM VCMMAE treatment. The relative cell viability rate fell further to approximately 20% with 200 nM of either 7DC2-VCMMAE or 7DC4-VCMMAE.

The cell surface expression of CD47 on A549 cells is approximately half of that on SPC-A-1 cells. A549 cultured with 200 nM 7DC2-VCMMAE or 7DC4-VCMMAE had a relative cell viability rate of only about 70% (**Figure 4C**). Together, these results suggest that the 7DC-VCMMAE ADCs have an excellently targeted therapeutic effect, particularly in cancer cells that express high levels of surface CD47 antigen.

### Phagocytosis of Targeted CD47 on NSCLC Cells

Next, we investigated whether the new ADCs are able to induce cancer cell phagocytosis by macrophages. In this experiment, cancer cells were co-cultured with macrophages prior to ADC treatment, but we found that even after 2.5 hours of co-culture, 95D or SPC-A-1 cells failed to adhere to the culture plate and remained suspended in the media. Only A549 cells attached to the culture plate with macrophages. Therefore, A549 cancer cells were chosen to evaluate the phagocytosis induction effect of the new ADCs *in vitro.*

**Figure 5:**
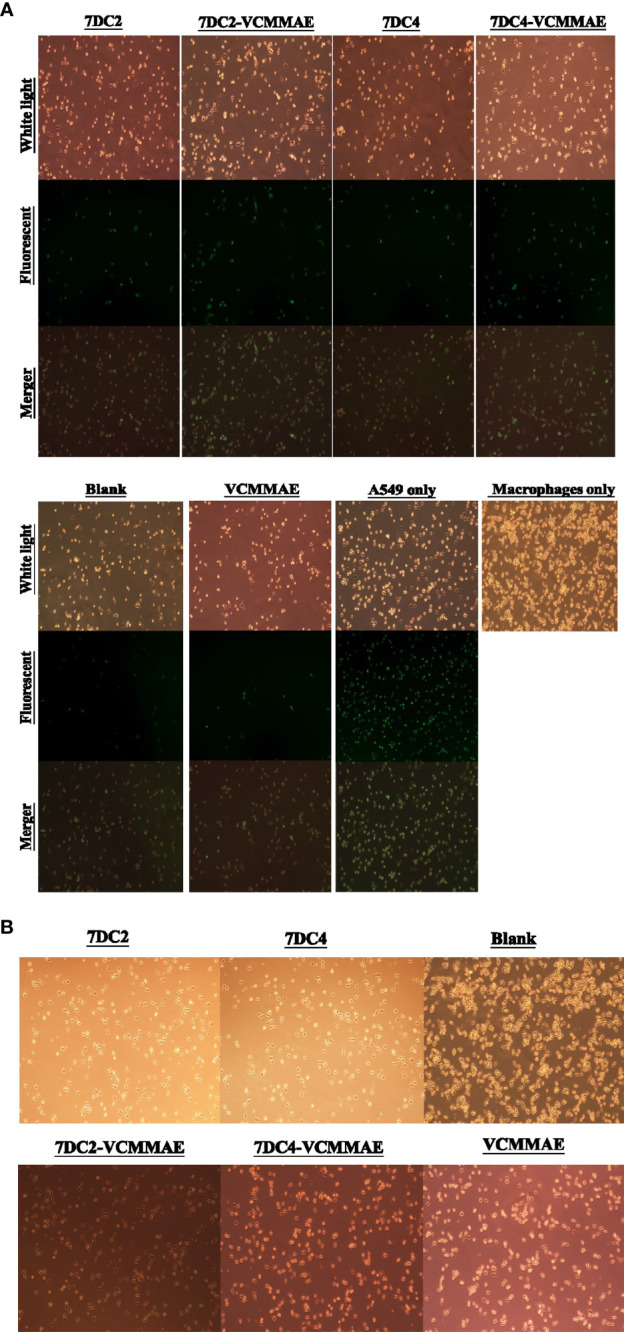
ADC targeting of CD47 induces phagocytosis of NSCLC cells. **(A)** Images of fluorescently labelled A549 cancer cell phagocytosis by macrophages. 7DC2-VCMMAE and 7DC4-VCMMAE elicit macrophage-mediated phagocytosis of A549 cancer cells through targeting CD47, supporting the idea that the ADCs inhibit the “don’t eat me” CD47 signal, increasing its antigenicity and promoting recognition by macrophages. **(B)** Cell morphology images of macrophages treated with 40 mM 7DC2 mAb, 7DC4 mAb, VCMMAE, 7DC2-VCMMAE, or 7DC4-VCMMAE, showing a shift to a more slender cell morphology suggesting macrophage activation compared with the blank group.

The effects of 7DC2, 7DC4, 7DC2-VCMMAE, and 7DC4-VCMMAE on macrophage-mediated phagocytosis of A549 cells labeled with CFDA SE (green) were observed by fluorescence microscopy. A549 cells were co-cultured with macrophages for 2.5 hours. The images in **Figure 5A** show that the labeled A549 cells have a circular cell morphology under light and fluorescence microscopy (green), whereas the unlabeled, migrating macrophages show extended filopodia and appear elongated under light microscopy. Co-cultures treated with media (blank) or VCMMAE showed a similarly small number of fluorescent A549 cells, indicating that the macrophages phagocytized A549 cells, but the low concentration of 40 nM VCMMAE had no additional cytotoxic killing effect. In the presence of 7DC2 or 7DC4 mAbs, phagocytosis was increased as the mAbs bound to CD47 on the surface of A549 cells and effectively inhibited the “don’t eat me” signal.

Interestingly, treatment with 40 nM of 7DC2-VCMMAE or 7DC4-VCMMAE significantly induced the phagocytosis of A549 cells. We speculate that 7DC2-VCMMAE and 7DC4-VCMMAE bind to CD47 on the A549 cell membrane and inhibit the “don’t eat me” signal, and that internalization of the VCMMAE payload causes cell damage that enhances macrophage recognition, thus greatly increasing phagocytosis. We also noted that macrophages treated with 40 mM of 7DC2, 7DC4, VCMMAE, 7DC2-VCMMAE, or 7DC4-VCMMAE appeared more slender compared with untreated cells (**Figure 5B**), suggesting activation of macrophages under these conditions.

### Antitumor Efficacy of ADC *In Vivo*

**Figure 6:**
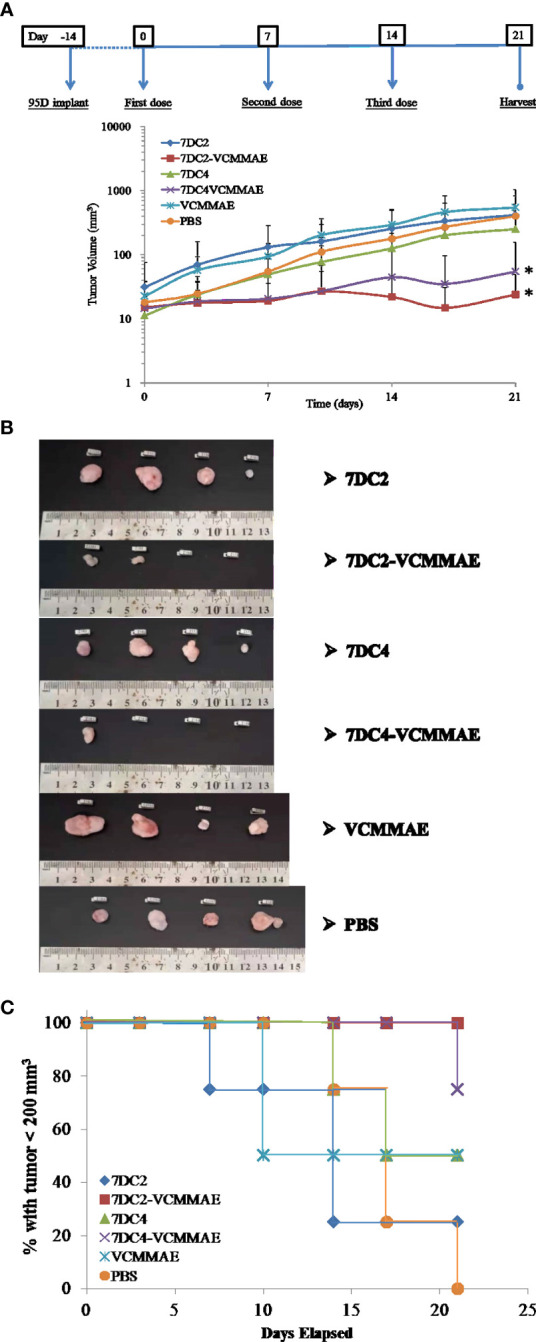
*In vivo* antitumor efficacy of the ADCs in a lung cancer cell-derived xenograft mouse model. **(A)** Changes in tumor size over time in xenograft mouse models bearing 95D tumors. Mice were randomly assigned into 6 groups and treated with 20 mg/kg of 7DC2-VCMMAE, 7DC4-VCMMAE, 7DC2, or 7DC4; 0.1755 mg/kg VCMMAE; or 200 μl PBS on day 0, 7, and 14. **(B)** Excised tumor tissues from each group mice on day 21 indicated greater antitumor efficacy of 7DC2-VCMMAE compared with 7DC4-VCMMAE. **(C)** Kaplan-Meier survival curves for the six treatment groups (defined by tumor size below 200 mm^3^, see methods). Mean values and standard deviations of all experiments are calculated from four independent measurements. * indicates P < 0.05.

To parallel our *in vitro* studies in human lung cancer cell lines, we evaluated the antitumor efficacy of our new ADCs *in vivo* using 95D cell line-derived xenografts (CDX) in an immunodeficient NOD/SCID mouse model. 95D cancer cells were implanted into NOD/SCID mice (day -14) and allowed grow for 14 days. Mice were then treated with 20 mg/kg 7DC2, 7DC4, 7DC2-VCMMAE, 7DC4-VCMMAE, or 0.1755 mg/kg free VCMMAE, and 200 μl PBS on days 0, 7, and 14, and xenograft tissues were harvested on day 21. 7DC2-VCMMAE or 7DC4-VCMMAE treatment almost completely eradicated the xenograft 95D tumor, with no signs of toxicity. 7DC2-VCMMAE was more effective than 7DC4-VCMMAE, and both ADCs were significantly more effective than the two 7DC mAbs or VCMMAE alone (**Figures 6A, B**). Representative images of the mice bearing 95D tumors and endpoint tumors harvested from xenograft models are shown in **Supplementary Figure S1** and **Figure 6B**.

Mice treated with 7DC2-VCMMAE and 7DC4-VCMMAE had higher survival rates, with significantly more mice surviving to the study endpoint (day 21), as shown in **Figure 6C**. All mice treated with 7DC2-VCMMAE survived, with treatment almost completely eradicating the xenograft tumors (**Figure 6B**). These results indicated that 7DC2-VCMMAE is more stable and effective than 7DC4-VCMMAE in targeting the xenograft tumors *in vivo*.

### Bio-Distribution of 7DC2-VCMMAE and 7DC4-VCMMAE

**Figure 7:**
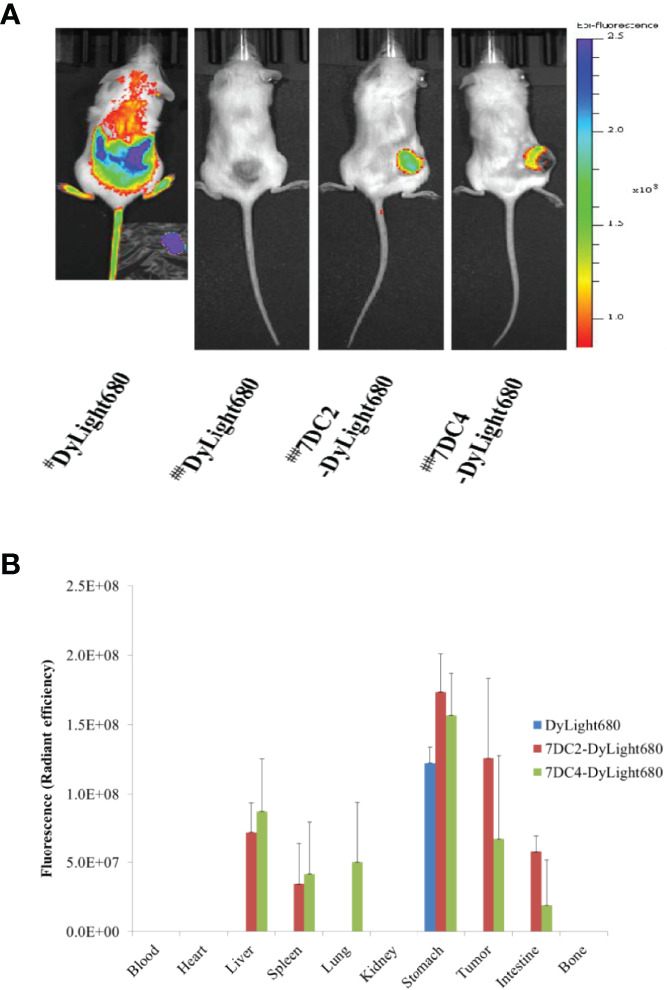
Bio-distribution of 7DC2-VCMMAE and 7DC4-VCMMAE in xenograft models. **(A)** *In vivo* fluorescence imaging of Dylight680, 7DC2-Dylight680, and 7DC4-Dylight680 in mice. # Indicates bio-distribution of the fluorescent marker 1 hour after injection; ## indicates distribution after 24 hours. **(B)** *Ex vivo* bio-distribution of Dylight680, 7DC2-Dylight680, and 7DC4-Dylight680 in the indicated organs or tumor tissue from 95D tumor-bearing mice at 24 hours post-injection (3 mg/kg) determined *ex vivo* with IVIS (see **Supplementary Figure S2**). The mean values and standard deviations are calculated from three independent measurements.

To further assess the targeting efficacy of 7DC2-VCMMAEand 7DC4-VCMMAE *in vivo*, we examined the bio-distribution of 7DC2-VCMMAE and 7DC4-VCMMAE in which VCMMAE was replaced with the small molecule fluorescent marker Dylight680 to form 7DC2-Dylight680 and 7DC4-Dylight680. The results of *in vivo* fluorescence imaging and *ex vivo* bio-distribution measurements are shown in **Figure 7**.

One hour after injection of free DyLight680 into mice, the fluorescent signal was distributed across a large area. After 24 hours, the fluorescent signal had completely disappeared, as free DyLight680 was degraded or metabolized. In contrast, both 7DC2-DyLight680 and 7DC4-DyLight680 fluorescent signals were localized at tumor sites 24 hours after administration (**Figure 7A**). Thus, we concluded that 7DC2-VCMMAE and 7DC4-VCMMAE likely localize to the 95D xenografts within one day after administration in our CDX NOD/SCID mouse model. Notably, a greater signal at the tumor site was seen with 7DC2-DyLight680 compared to 7DC4-DyLight680 (**Figure 7A**), further suggesting that 7DC2-VCMMAE is more stable and specific than 7DC4-VCMMAE in targeting CD47-expressing tumor tissues in mice.

In addition, we performed *ex vivo* NIRF imaging of organs harvested from the 95D tumor-bearing mice, as shown in **Figure 7B** and **Supplementary Figure S2**. One day after intraperitoneal injection of either 7DC2-VCMMAE or 7DC4-VCMMAE, fluorescent signals were detectable in the stomach tissue of mice in each group. No signals were detected in blood, heart, and bone, indicating that neither 7DC4-VCMMAE nor 7DC2-VCMMAE affects the hematopoietic system. The off-target distribution in liver and spleen was greater for 7DC4-DyLight680 than 7DC2-DyLight680, and 7DC4-DyLight680 only was detected in lung. 7DC4-DyLight680 and 7DC2-DyLight680 distribution to the intestine was observed, with greater signals from 7DC2-DyLight680 than 7DC4-DyLight680. Because the fluorescent signal in the intestine was probably produced by metabolites, these results indicate a faster metabolism and excretion rate of 7DC2-VCMMAE than 7DC4-VCMMAE.

Taken together, quantitative *ex vivo* measurement of bio-distribution showed that 7DC2-DyLight680 targeted the 95D tumor with high local concentration and low off-target distribution (**Figure 7B** and **Supplementary Figure S2**), which may underlie the observed higher antitumor potency of 7DC2-VCMMAE (**Figure 6**). Conversely, the higher off-target distribution of 7DC4-DyLight680 could explain the lower stability and specificity of 7DC4-MMAE in our xenograft model (**Figure 7**).

### *In vivo* Biosafety Analysis

We assessed serum biochemical markers and weight changes to evaluate the impact of drug off-target distribution and biological safety of the 7DC4-VCMMAE and 7DC2-VCMMAE ADCs. Though our bio-distribution analysis indicated that 7DC4 or 7DC2 localized to the liver (**Figure 7B** and **Supplementary Figure S2**), no biomarkers indicated hepatotoxicity, and there was also no signal of nephrotoxicity (**Table 2**). Body weight of mice in 7DC4-VCMMAE group decreased slightly after the first administration, but no significant change in body weight was noted in the other treatment groups (**Supplementary Figure S3**). Although both 7DC4 and 7DC2 distribute to the stomach after administration, 7DC2-VCMMAE and 7DC4-VCMMAE did not appear to cause gastric toxicity that would affect appetite or food consumption leading to weight loss. Therefore, the novel ADCs appear to be safe and tolerable in mice.

**Table 2:** Serum biomarkers of toxicity of ADC 7DC2-VCMMAE in mice*.

	ALT(U/L)	ALP(U/L)	BUN(mg/dL)	CRE(mg/dL)
7DC2	26.75 ± 5.80	88.00 ± 8.04	7.67 ± 0.81	10.73 ± 1.43
7DC2-VCMMAE	25.00 ± 2.45	104.50 ± 25.04	7.55 ± 0.94	15.15 ± 1.10
7DC4	43.75 ± 15.26	85.75 ± 4.50	7.36 ± 0.61	10.85 ± 3.80
7DC4-VCMMAE	22.75 ± 2.87	82.50 ± 5.26	7.60 ± 1.06	15.40 ± 2.51
VCMMAE	26.75 ± 2.87	72.00 ± 11.69	7.57 ± 0.59	14.00 ± 4.82
PBS	29.25 ± 4.99	72.25 ± 4.57	7.81 ± 0.34	10.03 ± 3.71

*Mean ± SD for n = 4 mice.

ALT, alanine aminotransferase; ALP, alkaline phosphatase; BUN, blood urea nitrogen; CRE, creatinine.

## Discussion

In this study, we developed and evaluated the efficacy and safety of first VC linker- based CD47-targeted ADCs with translational potential for the treatment of NSCLC. We clearly analyze, identify and describe the physicochemical properties and development process of our VC linker-based CD47-targeted ADCs, although anti-CD47-DM1 based on Sulfo-SMCC linker for treating triple-negative breast cancers was reported by Si, et al., in August 2021 (36). Additionally, larger precipitation would be occurred during constructing SMCC linker-based ADC according to the research report of Chiang, et al., in 2020, which greatly increased the production cost (42). And it cannot improve the drug loading of the antibody due to the steric effect, so its efficacy in cancer cells killing is less than the VC linker-based ADC (42). Furthermore, we demonstrated that 7DC2-VCMMAE has a great antitumor efficacy and specificity and both ADCs appear to be safe and tolerable in a xenograft mouse model of lung cancer.

SGN-35 (brentuximab vedotin, Adcetris^®^) has been approved by FDA in 2011, which is a VC linker-based ADC targeting CD30, having 4 DAR and possessing good tolerance in clinical use (38). It is aimed at non-solid tumors (Hodgkin’s lymphoma and anaplastic large-cell lymphoma), so it cannot achieve the localized effect and cause freer SGN35 to excurse in the blood. However, our 7DC-VCMMAE targets at solid tumors that can attract more ADCs from the blood into tumor tissues and localize the acting range of ADCs. Our studies suggest that the maximum tolerated dose (MTD) of 7DC-VCMMAE may be greatly increased. Additionally, solid tumors are usually more refractory than non-solid tumors, so higher doses are required. When the DAR of ADC is increased, the usage of ADC can be reduced and its targeted anti-tumor efficacy and safety can all be improved. Animal experiments for treating solid tumor model, usually show that the best anti-tumor dose is 30 mg/kg for CDX-mice model (Kuo, et al., 2019, MABS) (43); however, 7DC-VCMMAE at 20 mg/kg for CDX-model mice show quite good anti-tumor effects.

Both 7DC2 and 7DC4 were able to penetrate lung cancer cells and had similar chemical and physical properties, such as affinity and hydrophobicity, yet we observed differences in their efficacy and specificity in our xenograft mouse model of lung cancer. LDS-PAGE indicated differences in the molecular weights of the two IgG1s, with the 7DC2-light chain molecular weight less than that of the 7DC4-light chain, and the 7DC2-heavy chain molecular weight more than that of the 7DC4-heavy chain (**Figure 2B**). Sequence differences between 7DC2 and 7DC4 may also alter their epitopes and glycosylation sites (**Supplementary Figure S4**), which could underlie observed differences in the stability and accuracy of each ADC *in vivo*.

We found that 7DC2-VCMMAE and 7DC4-VCMMAE have greater cytotoxic effects against 95D and SPC-A1 cells compared to A549 cells. This may be due to the lower expression of CD47 on the A549 cell membrane, suggesting that 7DC-VCMMAE may have a greater therapeutic effect for tumors that express high levels of CD47 antigen. Additionally, we found that VCMMAE alone at a concentration of less than 200 nM does not effectively kill the cancer cells, likely because it is unable to specifically enter the cancer cells. Conjugation of VCMMAE to our 7DC antibodies at a high DAR allowed the drug to specifically enter the cancer cells and kill them at a lower effective concentration. Furthermore, our study suggests that the new ADCs have a two-pronged anti-cancer effect, as our cell phagocytosis experiments found that both ADCs have the ability to promote macrophage-mediated phagocytosis of A549 cancer cells, and 7DC2-VCMMAE seems to have a greater effect than 7DC4-VCMMAE.

A549 cancer cells can synthesize lecithin containing a high concentration of unsaturated fatty acids, the building blocks of phospholipids. Phospholipid is one of the main components of cell membranes and plays an important role in the division of organelles, protein storage in cell signal transduction, cell adhesion, and cell cycle regulation (44). Furthermore, phospholipids are involved in tumor cell proliferation, migration, adhesion, apoptosis, signal transduction, cell cycle regulation, and other activities (44). We speculate that alterations in phospholipid synthesis may be one of the reasons why A549 is relatively insensitive to 7DC-VCMMAE, as ADCs depend on cell membrane receptor-mediated endocytosis to enter the tumor cell. Another possibility is that autophagy of A549 cells treated with ADCs may decrease the cytotoxic efficacy of the ADCs. For tumor cells such as A549, the ability of CD47-specific ADCs to stimulate phagocytosis may overcome the limitation of insufficient cytotoxicity.

For targeted therapy *in vivo*, we confirmed that 7DC-VCMMAE can effectively inhibit tumor growth, consistent with the results of our *in vitro* cytotoxicity experiments. Furthermore, 7DC2-VCMMAE was more effective than 7DC4-CVMMAE in the xenograft tumor models (**Figure 6**). The survival rate of mice in the 7DC4-CVMMAE group was lower than that of mice in the 7DC2-VCMMAE group, likely due to differences in stability and specificity between the two targeting antibodies. Bio-distribution analysis showed that 7DC2-VCMMAE was more stable and targeted tumor tissue with greater specificity *in vivo* compared to 7DC4-VCMMAE. While safety and tolerability signals were similar for both ADCs, we conclude that 7DC2-VCMMAE has good potential for further development as a novel therapeutic for NSCLC.

## Data Availability Statement

The original contributions presented in the study are included in the article/**Supplementary Material**. Further inquiries can be directed to the corresponding authors.

## Ethics Statement

All mouse experiments were conducted according to guidelines and experimental protocols approved by the Institutional Animal Care and Utilization Committee (IACUC) of Fujian Normal University (Protocol ID: 20200010).

## Author Contributions

Data curation: Z-CC. Formal analysis: Z-CC. Investigation: Z-CC. Resources: SF, Y-KS, HW, DW, YZ. Validation: QC, JL. Writing – original draft: Z-CC. Writing – review and editing: JL, QC. FACS data curation: DC. All authors contributed to the article and approved the submitted version.

## Funding

This work was supported by the Strait Postdoctoral Exchange Funding Program, Fujian Province, China (Grant no. 2019A001).

## Conflict of Interest

The authors declare that the research was conducted in the absence of any commercial or financial relationships that could be construed as a potential conflict of interest.

## Publisher’s Note

All claims expressed in this article are solely those of the authors and do not necessarily represent those of their affiliated organizations, or those of the publisher, the editors and the reviewers. Any product that may be evaluated in this article, or claim that may be made by its manufacturer, is not guaranteed or endorsed by the publisher.

## Supplementary Material

Supplementary Figure 1 Tolerance of the mice to 7DC2-VCMMAE or 7DC4-VCMMAE treatment. 95D cancer cells were implanted into NOD/SCID mice (day -14) and allowed grow for 14 days. The mice treated with 20 mg/kg 7DC2, 7DC4, 7DC2-VCMMAE, 7DC4-VCMMAE, or 0.1755 mg/kg free VCMMAE, and 200 μl PBS on days 0, 7, and 14, and xenograft tissues were harvested on day 21. The images showed live mice, anesthesia, bearing 95D tumors on day 21, before harvesting tumors.

Supplementary Figure 2
*Ex vivo* NIRF imaging of organs from 95D tumor-bearing mice. Representative images of organs from 95D tumor-bearing mice 24 hours after intraperitoneal injection of DyLight 680, 7DC2-DyLight680, or 7DC4-DyLight680. 1. Blood, 2. Heart, 3. Liver, 4. Spleen, 5. Lung, 6. Kidney, 7. Stomach, 8. Tumor, 9. Intestine, 10. Bone.

Supplementary Figure 3 Body weight changes after administration of ADC to assess biosafety. There were no significant changes in the body weight of mice in any of the treatment groups, with the exception of a small decrease on day 3 in mice treated with 7DC4-VCMMAE that resolved by day 7, indicated by the asterisk (P<0.05).

Supplementary Figure 4 Alignment and predicted N-glycan patterns of 7DC2 and 7DC4. 7DC2 and 7DC4 have slightly different amino sequences in the heavy and light chains, and 7DC4 has a glycosylation site indicated by a blue box, within its heavy chain variable region. Asterisks indicate overlapping residues within the two sequences.

Supplementary Table 1 Cell Surface Expression of CD47 on Different Lung Cancer Cell Lines. The CD47 molecules on the cell membranes of A549, SPC-A-1 and 95D cells were bound to 7DC and 7DC-VCMMAE, respectively, and then interacted with the goat anti-Human IgG Fc Secondary Antibody labeled with PE. Mean Fluorescence Intensity (MFI) obtained by using flow cytometry was employed to compare the expression of CD47 differently in three cancer cell lines with different properties. Events (Cell count) were 5000 cells per group for calculating.

## Abbreviations

ADCs, antibody-drug conjugates; CD47, cluster of differentiation 47; CDX, cell line-derived xenograft; DAR, drug-to-antibody ratio; EC_50_, half-maximal effective concentration; IC_50_, half-maximal inhibitory concentration; LDS, lithium dodecyl sulfate; NOD/SCID, nonobese diabetic/severe combined immunodeficient; NSCLC, non-small cell lung cancer; NIRF, near-infrared fluorescence; TCEP, Tris(2-carboxyethyl)phosphine; VCMMAE, Valine-citrulline-monomethyl auristatin E.
