# Supplementary figures and images for: Development of Novel CD47-Specific ADCs Possessing High Potency Against Non-Small Cell Lung Cancer in vitro and in vivo
Source: Front Oncol. 2022 May 12;12:857927. doi: 10.3389/fonc.2022.857927 (PMC9133542; doi:10.3389/fonc.2022.857927)

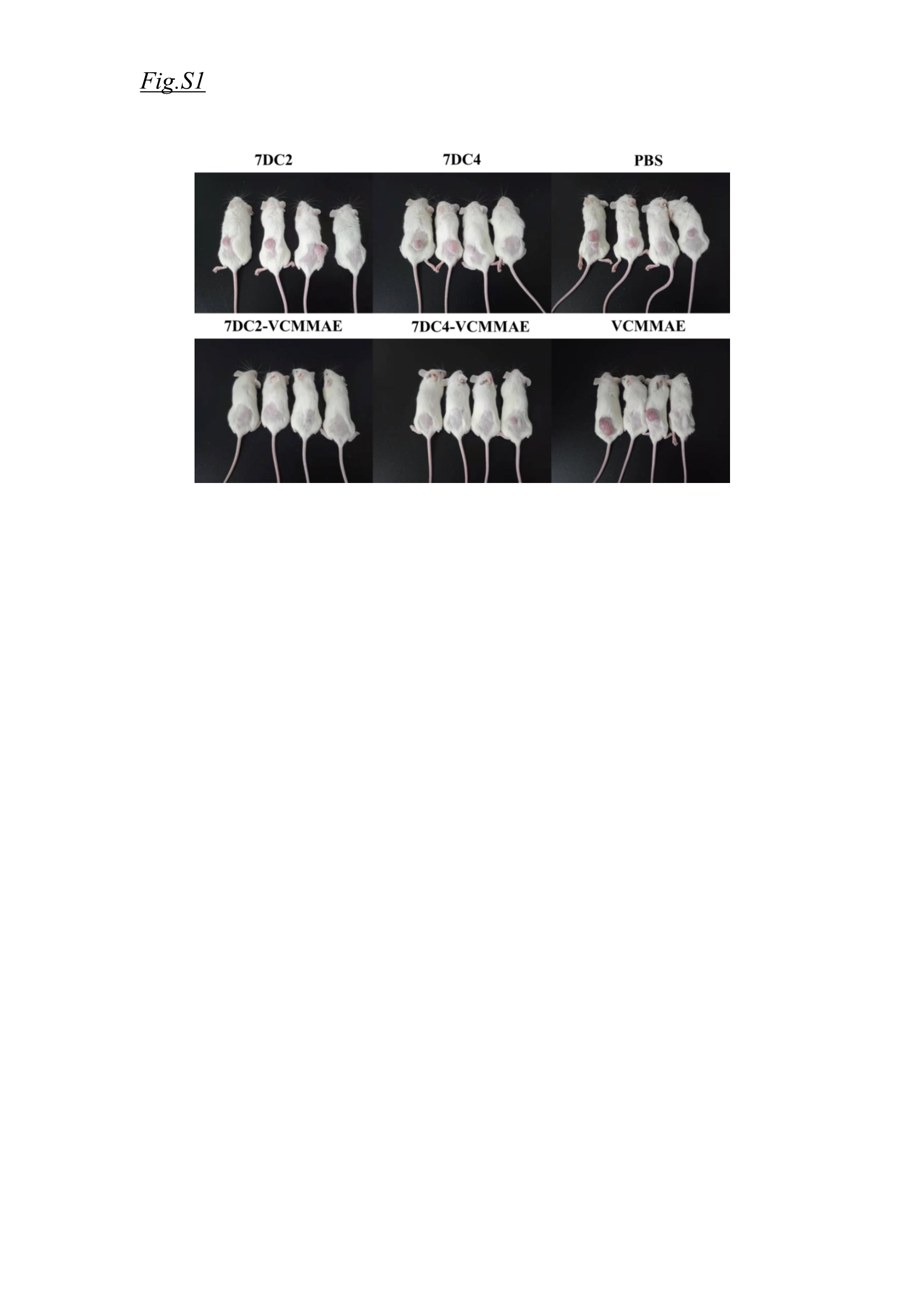

Supplement: Supplementary Figure 1 — Tolerance of the mice to 7DC2-VCMMAE or 7DC4-VCMMAE treatment. 95D cancer cells were implanted into NOD/SCID mice (day -14) and allowed grow for 14 days. The mice treated with 20 mg/kg 7DC2, 7DC4, 7DC2-VCMMAE, 7DC4-VCMMAE, or 0.1755 mg/kg free VCMMAE, and 200 μl PBS on days 0, 7, and 14, and xenograft tissues were harvested on day 21. The images showed live mice, anesthesia, bearing 95D tumors on day 21, before harvesting tumors. [file Image_1.tiff]

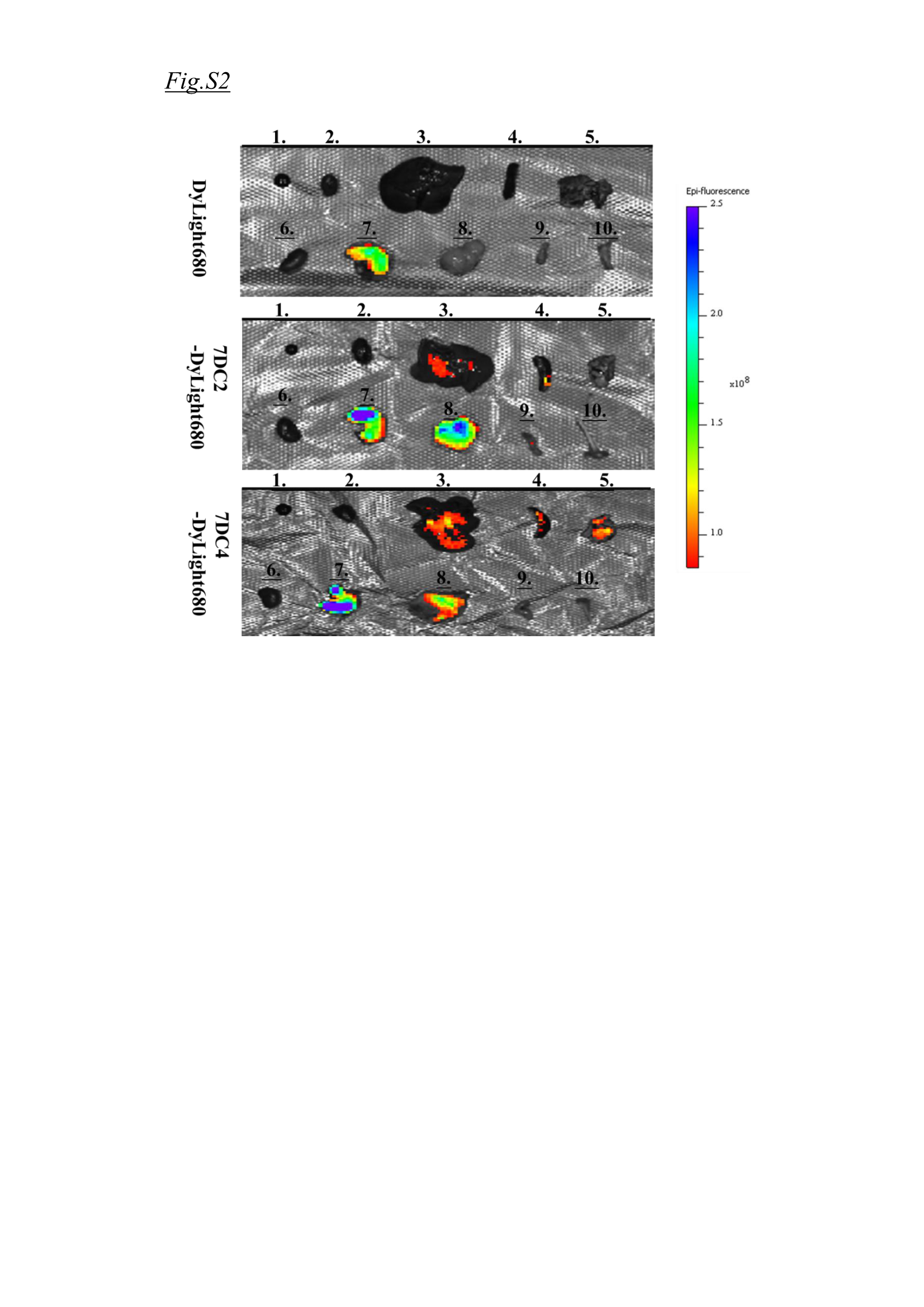

Supplement: Supplementary Figure 2 — Ex vivo NIRF imaging of organs from 95D tumor-bearing mice. Representative images of organs from 95D tumor-bearing mice 24 hours after intraperitoneal injection of DyLight 680, 7DC2-DyLight680, or 7DC4-DyLight680. 1. Blood, 2. Heart, 3. Liver, 4. Spleen, 5. Lung, 6. Kidney, 7. Stomach, 8. Tumor, 9. Intestine, 10. Bone. [file Image_2.tiff]

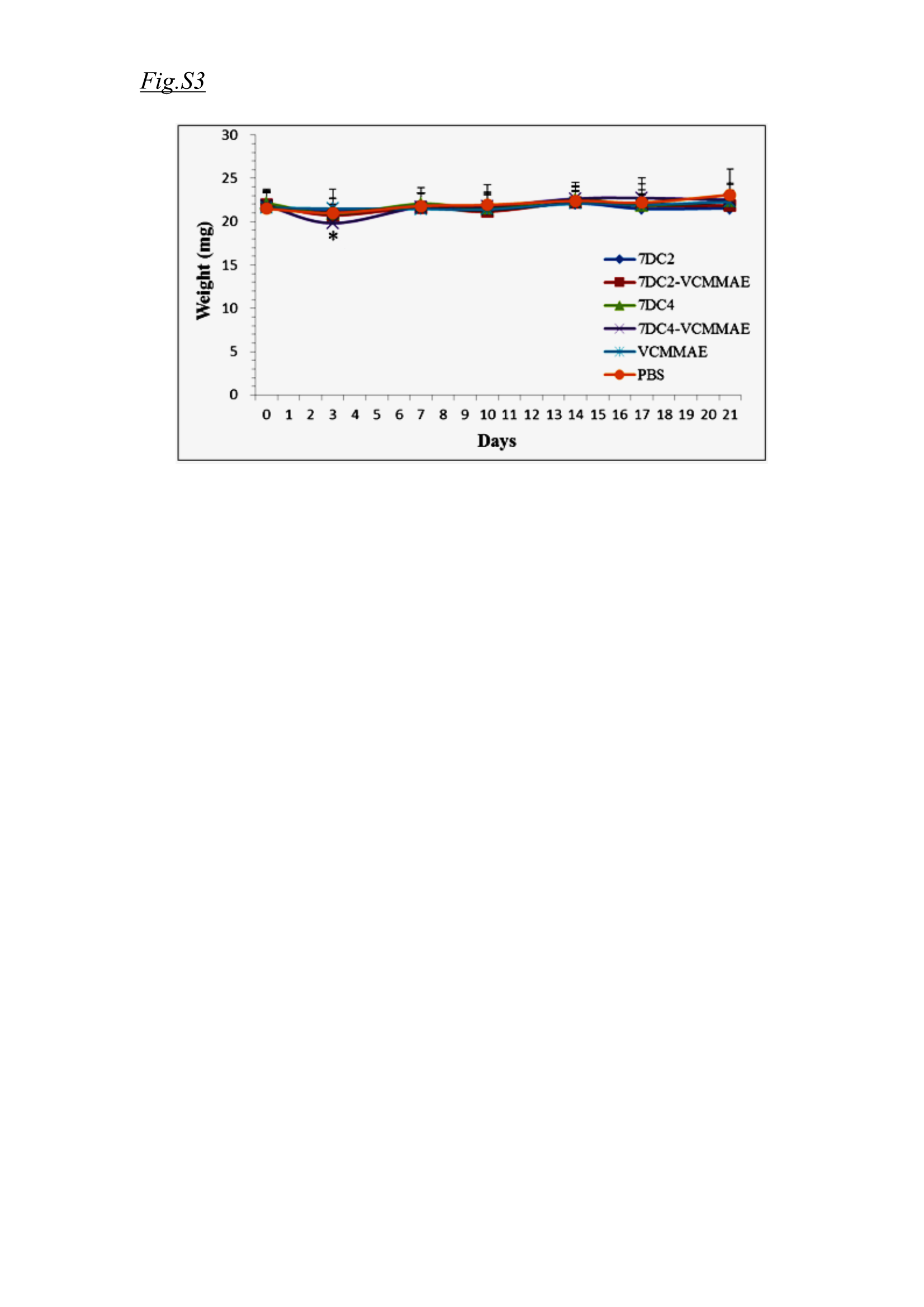

Supplement: Supplementary Figure 3 — Body weight changes after administration of ADC to assess biosafety. There were no significant changes in the body weight of mice in any of the treatment groups, with the exception of a small decrease on day 3 in mice treated with 7DC4-VCMMAE that resolved by day 7, indicated by the asterisk (P<0.05). [file Image_3.tiff]

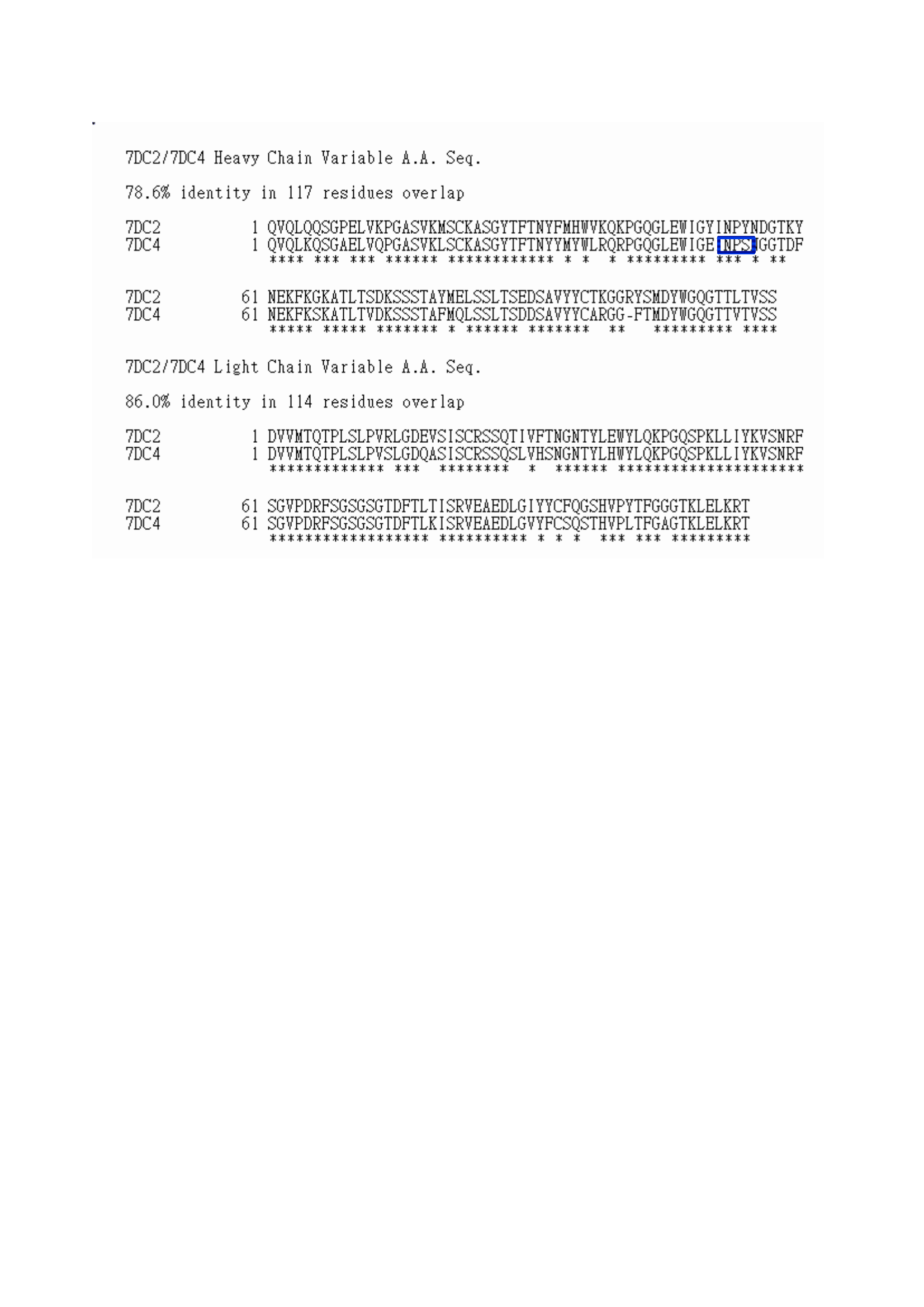

Supplement: Supplementary Figure 4 — Alignment and predicted N-glycan patterns of 7DC2 and 7DC4. 7DC2 and 7DC4 have slightly different amino sequences in the heavy and light chains, and 7DC4 has a glycosylation site indicated by a blue box, within its heavy chain variable region. Asterisks indicate overlapping residues within the two sequences. [file Image_4.tiff]

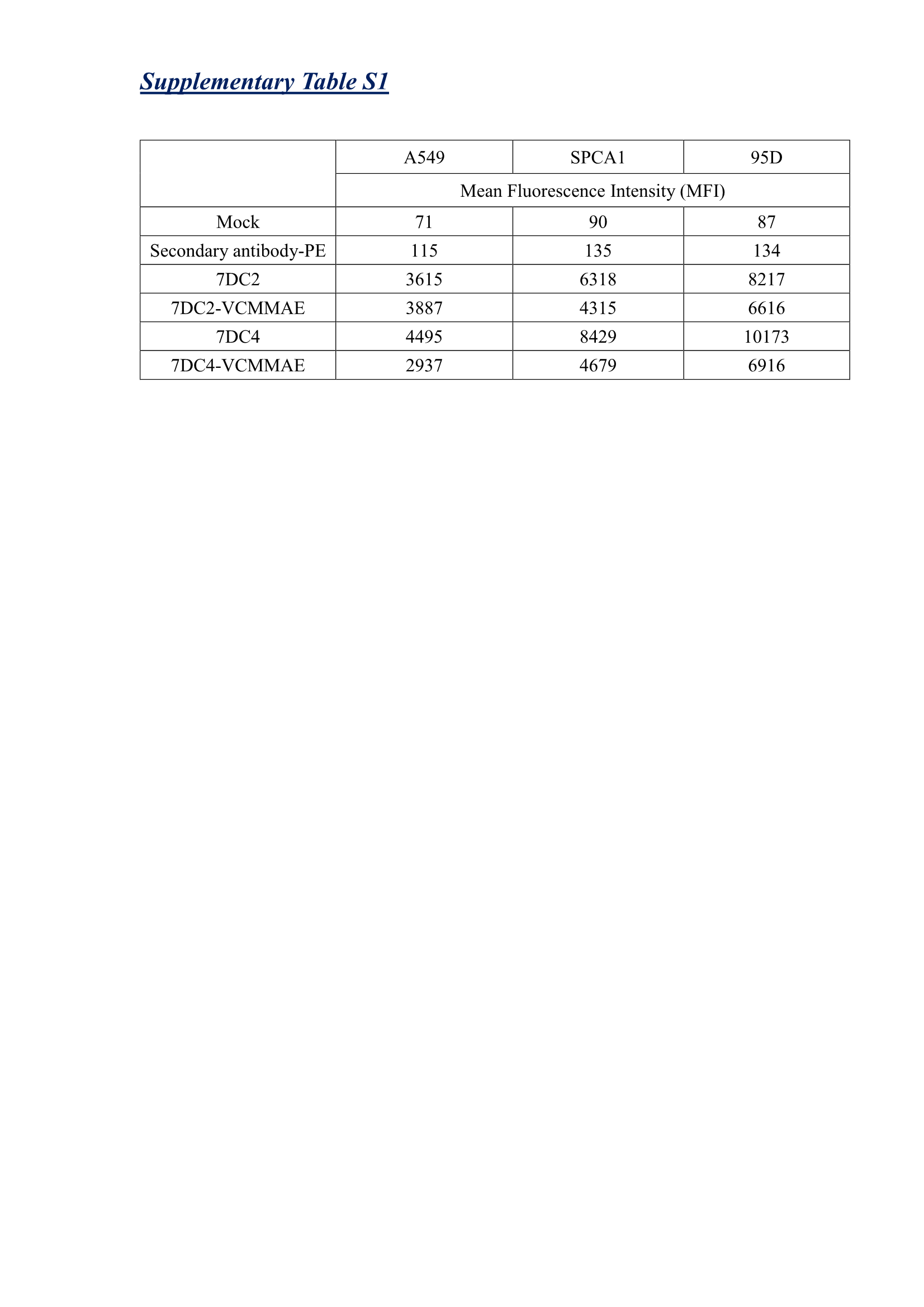

Supplement: Supplementary Table 1 — Cell Surface Expression of CD47 on Different Lung Cancer Cell Lines. The CD47 molecules on the cell membranes of A549, SPC-A-1 and 95D cells were bound to 7DC and 7DC-VCMMAE, respectively, and then interacted with the goat anti-Human IgG Fc Secondary Antibody labeled with PE. Mean Fluorescence Intensity (MFI) obtained by using flow cytometry was employed to compare the expression of CD47 differently in three cancer cell lines with different properties. Events (Cell count) were 5000 cells per group for calculating. [file Image_5.tiff]
